# Establishment and Validation of a Two-Step LAMP Assay for Detection of *Bacillus cereus*-Group Isolates in Food and Their Possibility of Non-haemolytic Enterotoxin Production

**DOI:** 10.3389/fmicb.2022.930648

**Published:** 2022-06-09

**Authors:** Annemarie Busch, Ulrich Schotte, Nadja Jeßberger, Hendrik Frentzel, Madeleine Plötz, Amir Abdulmawjood

**Affiliations:** ^1^Institute for Food Quality and Food Safety, University of Veterinary Medicine Hannover, Hanover, Germany; ^2^Department A (Veterinary Medicine), Central Institute of the Bundeswehr Medical Service Kiel, Kronshagen, Germany; ^3^Unit Bacterial Toxins, Food Service, Department of Biological Safety, German Federal Institute for Risk Assessment, Berlin, Germany

**Keywords:** LAMP, *Bacillus cereus*, *nheB*, *groEL*, toxin production, fast detection

## Abstract

The closely related members of the *Bacillus cereus*-group can mainly only be differentiated by whole genome sequencing. Among them, there are potentially toxin-producing bacteria. When consumed with food, these can cause vomiting or diarrhea and abdominal cramps. To date, although no EU-wide threshold exists, a bacterial count of 10^5^ CFU/g can be regarded as critical. Specific and rapid detection of the bacteria is difficult due to their close relationship, and no loop-mediated isothermal amplification (LAMP) assay has been developed so far to detect potentially toxin-producing members of the *B. cereus*-group. Aim of this study was to develop a LAMP method to detect critical cell counts specifically and rapidly of potentially non-haemolytic enterotoxin (NHE)-producing cells of this group. A two-step LAMP assay was developed. First, the target sequence *groEL* was used to determine the representatives of the *B. cereus*-group. Second, since bacteria in which *nheB* is present are basically capable of producing enterotoxins, this gene was chosen for detection. The specificity of the developed assay was 100% for *B. cereus*-group isolates and 93.7% for the detection of *nheB*. The analytical sensitivity was 0.1 pg DNA/μl. Using simplified DNA extraction by boiling, cell-based sensitivity was determined. Targeting *groEL* and *nheB*, 11.35–27.05 CFU/reaction and 11.35–270.5 CFU/reaction were detectable, respectively. Artificially contaminated samples were investigated to prove the application in foods. Direct detection of the critical value of *B. cereus*-group cells was possible in 83.3% of samples and detecting the toxin-gene 50% thereof. After a 6-h incubation period, the detection rate increased to 100 and 91.7%, respectively. Additionally, 100 natively contaminated food samples were tested, also quantitatively and culturally. Samples with relevant contamination levels were reliably detected using *groEL*-LAMP. After a 6-h incubation period, isolates bearing the toxin gene *nheB* could also be reliably detected. In addition, colony material was boiled and used as a LAMP template for simple detection. Specificity for the *B. cereus*-group was 100 and 93.22% detecting *nheB.* The study demonstrated that screening of food samples with the *groEL*/*nheB*-LAMP assay can be performed within 1 day, making it possible to detect critical levels of potentially NHE-toxin-producing cells of the *B. cereus*-group.

## Introduction

*Bacillus cereus* (*B. cereus*) is a spore-forming and potentially toxin-producing bacterium. *B. cereus* is the eponym of the so-called *B. cereus* group. The *B. cereus* group consists of 17 closely related species, of which especially those added in 2017 can only be distinguished by whole genome sequencing (WGS) (Liu et al., 2017). The species *B. weihenstephanensis* is considered synonymous with *B. mycoides* and was therefore renamed, but both names remain common (Liu et al., 2018; Parte et al., 2020). Further changes in group membership are expected (Carroll et al., 2020). Synonyms for the *B. cereus* group are *B. cereus sensu lato* (*s.l*.) and presumptively *B. cereus*. The latter term is often used in DIN EN ISO standards where no clear differentiation between the representatives of the group is possible with the specified tests. A detection method that refers exclusively to *B. cereus* is therefore difficult to implement.

*B. cereus* entries into the food chain are possible due to its ability to form biofilms, but most frequently, it is transferred to foods through the contamination of raw foodstuffs. Heat treatment can kill vegetative cells. Nevertheless, *B. cereus* can survive this treatment in the form of spores and germinate again under favorable environmental conditions. Therefore, complete prevention of contamination is virtually impossible. Bacterial toxins are a common component in cases of food poisoning. *B. cereus* toxins are among the most important triggers of food poisoning and thus play an important role in food safety (Dietrich et al., 2021). Both bacteria and toxins can be ingested when contaminated food is consumed. As an approximation, a bacterial count of 10^5^ colony-forming units (CFU)/g food is sufficient to produce relevant toxin quantities directly in the consumed food or in the small intestine, while lower levels (100–1,000 CFU/g) are usually tolerated (Rajkovic et al., 2008; Stenfors Arnesen et al., 2008; BfR, 2020a,b; Dietrich et al., 2021). However, cases have also been reported where fewer cell counts were sufficient to cause disease symptoms (Jovanovic et al., 2021).

Two types of disease can be distinguished. One type is the slightly more frequently occurring emetic disease (Jessberger et al., 2020). Here, a toxin (cereulide) preformed in the food during the proliferation of vegetative cells is ingested, which is resistant to heat, acid, and proteolysis (Rajkovic et al., 2008). In addition, spores for the most part and some vegetative cells might be consumed. Intoxication leads to nausea and vomiting within 0.5–6 h after consumption. The symptoms usually subside quickly. Very rarely are there severe courses of disease resulting in liver and brain damage (Dierick et al., 2005; Shiota et al., 2010). The other type of disease is characterized by diarrhea and abdominal cramps. In this case, *B. cereus* spores are predominantly ingested. The spores germinate in the small intestine and form enterotoxins as vegetative cells. These forms (vegetative cells and enterotoxins) are sensitive to heat, acid and proteases. Ingested vegetative cells as well as enterotoxins formed in the food are inactivated during the gastrointestinal passage. Thus, the incubation period of diarrhoeal disease is 6–24 h (EFSA, 2005; Griffiths and Schraft, 2017). The diseases are often associated with the consumption of starchy, cooked foods (rice, pasta, etc.). However, recent studies have shown that products such as vegetables, fruit, sauces, salads as well as meat and meat products are also affected by contamination (Jessberger et al., 2020; Dietrich et al., 2021). However, the killing of and thus reduction in the number of cells by heat treatment supports the germination of the spores and the multiplication of the vegetative cells if this process does not take place fast enough or with sufficiently high temperatures. Therefore, sufficient, and rapid cooling (≤7°C) or maintaining the temperature (≥60°C) after heat treatment is necessary to prevent growth of *B. cereus* (BfR, 2020b). Diagnostic approaches for differentiation in this closely related group by nucleic acid amplification like polymerase chain reaction (PCR) and loop-mediated isothermal amplification method (LAMP) are available. However, these are very challenging to implement due to the heterogeneity of the group. Therefore, the focus of other studies has often been on the detection of specific toxin genes or directly on the production of the protein (Liu et al., 2010; Abdulmawjood et al., 2019) or selecting several target regions that are specific, too, for example, the *B. cereus* group (Lim et al., 2011; Wei et al., 2018). A LAMP assay for detecting the genetic prerequisites to produce cereulide has already been established based on the pCER270 plasmid-located *cesA* (Liu et al., 2010). Also, a multiplex LAMP approach exists for simultaneous detection of *B. cereus* and *Staphylococcus aureus* (Deng et al., 2019).

The aim of the study carried out here was to develop a rapid and reliable method for detecting relevant *B. cereus* and correspondingly high cell contents in food using the LAMP method. For this purpose, a basic determination of potentially toxin-forming cells with correspondingly risky cell contents in the food should be directly detected. In the further course of the examination of the food, it should then be determined whether the representatives of the detected *B. cereus* group also carry enterotoxin-genes. This approach offers the distinct advantage over the microbiological reference method that results can be delivered more quickly and reliably.

## Materials and Methods

### Target Gene Selection and Primer Design

As potential *B. cereus*-group-specific target genes, *groEL* (Park et al., 2007; Lim et al., 2011) and *gyrB* (La Duc et al., 2004; Dzieciol et al., 2013; Wei et al., 2018) were included in this study. All gene sequences were checked by BLAST analysis using the database of the National Centre for Biotechnology Information.^1^ The BLAST analysis included 50 *B. cereus* isolates, 50 *B. cereus* group and 50 non-*B. cereus* group strains. Primers (F3/B3, FIP/BIP, LoopF/LoopB) were created for all target genes using the free available software Primer Explorer V5 (Fujitsu Limited).^2^ Mutants within the primers targeting group-specific genes (*groEL, gyrB*) were identified by BLAST analysis and labeled according to the IUPAC code. In the second step, one of the *nhe* genes (*nheB*) was chosen because they are present in nearly all enteropathogenic *B. cereus* strains, as described by Jeßberger et al. (2015). *NheB* encodes a protein component of the non-haemolytic enterotoxin complex (NHE). In addition, the amount of NheB protein correlates with cytotoxicity. For this gene, primers were designed without respective mutants after BLAST analysis. Primers with respective mutants (degenerate) and without (non-degenerate) were ordered from Eurofins Genomics GmbH (Ebersberg, Germany).

### Reaction Approach and Preliminary Test

All LAMP reactions were performed using the Genie^®^ II portable real-time fluorometer (OptiGene Ltd., Horsham, United Kingdom). The assay protocol used followed the manufacturer’s recommendations. The amplification took place at 65°C for 40 min. The melting curve was generated in a temperature range of 98°C–80°C (ramp rate = 0.05°C/s). Individual primers from each target-specific primer set were combined into a primer mix at a standard concentration recommended by the manufacturer OptiGene (F3/B3 0.2 μM, FIP/BIP 0.8 μM, LoopF/LoopB 0.4 μM). Each 25 μl LAMP reaction batch contained 15 μl GspSSD isothermal master mix ISO-001 (AmplexDiagnostics GmbH, Gars Bahnhof, Germany), 2.5 μl primer mix, 2.5 μl nuclease-free water and 5 μl DNA at a concentration of 0.1 ng/μl. Thus, it contained a total volume of 25 μl. In each run, one reaction batch with 5 pg DNA of *B. cereus* DSM 31 was used as positive control and one reaction batch without template as negative control (NTC).

In a first preliminary test, all primer sets prepared for determining the *B. cereus* group were tested using five isolates of the *B. cereus* group and two non-*B. cereus* group strains. In the second preliminary experiment, the remaining primer sets were tested on five further *B. cereus* isolates and three further *B. cereus* group isolates. The primer set for detecting the toxin gene *nheB* was tested on four isolates of the *B. cereus* group, three of which were carriers of the toxin gene. Additionally, one *Bacillus* sp. and two non-*Bacillus* strains were tested.

Used primers are shown in Table 1.

**TABLE 1:** *groEL* and *nheB* based primer sequences used for establishing the LAMP assay.

Primer	Sequence 5′→3′	Region*
***groEL:*** Acc.-No.: NC_004722 (region: 257826…259460)		
F3	GAAGAAGCACGTCGTTCG	25–42
B3	TTTTGCTAGCAACTTCTGC	220–238
FIP (F1c + B2)	AGTGGTGAACCAAATTTTTTCTCAA- ACTCTTGCAAACGCAGTA	116–140; 61–78
BIP (B1c + B2)	AATGATGGTGTAACAATCGCAAAAG- CTAATTTCGCACCCATGTT	148–172; 199–217
LF	TGGTCCAAGCGTAACTTT	79–96
LB	TCGAATTAGAAGATGCATTCG	176–196
***nheB:*** Acc.-No.: NC_004722 (region 1766446…1767654)		
F3	CTATTATGATACTTTAGTTGCTGC	423–446
B3	CGTTGTAATTTGATTTTGCAGAAG	649–672
FIP (F1c + B2)	CTGATCCACTTGCGCTTTATTTTCA-TAAAGCGACTCTTACGAAAGG	510–534; 462–482
BIP (B1c + B2)	CCGAAATAAAATGACTTCGGATACG-TCCTGCATCTTGACTAGC	558–582; 625–642
LF	CTACTTGATAATCTTGTTAAG	483–503
LB	CAAAACTTCAAGGGTGAT	583–600

*In chosen Sequence. Bold values for highlighting the genname.

### Bacterial Strains and Culture Conditions

A total of 83 *B. cereus* group isolates with the toxin gene and 13 without, including *B. cereus*, were used in this study. A total of 44 non-*B. cereus* group strains were selected, based on close genetic relationship to the *B. cereus* group or their importance for food safety. All used isolates are shown in Table 2. All strains were grown on Columbia agar with 5% sheep blood Plus (COLS) (Oxoid Deutschland GmbH, Wesel, Germany) at 37°C for 24 h under aerobic conditions. Every strain used was confirmed by MALDI-TOF analysis using the LT/SH device (Bruker Corporation, Bremen, Germany). All *Bacillus* spp., *Staphylococcus* spp. and *Listeria* spp. strains were prepared with the ethanol and formic acid extraction method (Schulthess et al., 2014; Bastin et al., 2019) before spotting on the target. The other strains were transferred directly.

**TABLE 2:** Strains used for specificity tests in this study.

Strain and strain ID	No. of strains	LAMP	
		
		*groEL*	*nheB*
***Bacillus cereus*** ** group (with *nheB-*toxin gene)**	**83**	**83**	**77**
*Bacillus anthracis*	1	1	1
*Bacillus cereus* (incl. ATCC 11778, DSM 1230, DSM 31, DSM 4384)	39	39	38
*Bacillus luti*	1	1	1
*Bacillus mobilis*	3	3	3
*Bacillus mycoides* (incl. CCUG 26678T)	3	3	3
*Bacillus paranthracis*	9	9	9
*Bacillus thuringiensis* (incl. CCUG 7429T)	5	5	5
*Bacillus thuringiensis* ssp. *aizawai*	2	2	2
*Bacillus thuringiensis* ssp. *israelensis*	1	1	1
*Bacillus thuringiensis* ssp. *kurstaki*	2	2	2
*Bacillus thuringiensis* ssp. *tenebrionis*	2	2	2
*Bacillus toyonensis* (incl. BCT-7112)	5	5	0
*Bacillus weihenstephanensis* (incl. CCUG 58725)	7	7	7
*Bacillus wiedmannii* (incl. DSM 102050)	3	3	3
***Bacillus cereus*** ** group (without *nheB*-toxin gene)**	**13**	**13**	**2**
*B. cereus*	1	1	0
*Bacillus cytotoxicus* (incl. DSM 22905)	2	2	0
*Bacillus mycoides*	1	1	0
*Bacillus pacificus*	1	1	1
*Bacillus paramycoides*	1	1	1
*Bacillus pseudomycoides* (incl. DSM 12442)	6	6	0
*Bacillus weihenstephanensis*	1	1	0
**Non-*B. cereus* group**	**44**	**0**	**0**
*Bacillus licheniformis*	10	0	0
*Bacillus pumilus* (incl. DSM 27)	2	0	0
*Bacillus subtilis* (incl. DSM 347)	5	0	0
*Campylobacter coli* (NCTC 12568)	1	0	0
*Enterobacter cloaecae* (NCTC 13464)	1	0	0
*Enterococcus faecalis* (DSM 13591)	1	0	0
*Escherichia coli* (DSM 1103)	1	0	0
*Lactobacillus casei* (DSM 20011)	1	0	0
*Lactococcus lactis* (DSM 20481)	1	0	0
*Listeria innocua* (incl. DSM 20649)	2	0	0
*Listeria ivanovii* (DSM 12491)	1	0	0
*Listeria monocytogenes* (CCUG 15526T, DSM 19094, NCTC 10527)	3	0	0
*Micrococcus luteus* (DSM 1790)	1	0	0
*Proteus mirabilis* (DSM 4479)	1	0	0
*Pseudomonas aeruginosa* (DSM 93*9)*	1	0	0
*Salmonella enterica* ssp. *enterica Ser. Enteritidis* (DSM 14221)	1	0	0
*Salmonella enterica* ssp. *enterica* Ser. *Typhimurium* (DSM 19587)	1	0	0
*Shigella flexineri* (DSM 4782)	1	0	0
*Shigella sonnei* (DSM 4782)	1	0	0
*Staphylococcus aureus* (ATCC 29213, DSM 1104, DSM 18597, DSM 799, NCTC 8325)	5	0	0
*Streptococcus thermophilus* (CCUG 21957)	1	0	0
*Yersinia enterocolitica* (DSM 11502)	1	0	0
*Yersinia pseudotuberculosis* (DSM 8992)	1	0	0

### DNA Extraction Method From Strains, Cell Suspensions and Enriched Food Matrices

DNA from strains was extracted using the DNEasy Blood and Tissue Kit (Qiagen GmbH, Hilden, Germany) according to the manufacturer’s protocol, and the DNA concentration in the eluate was determined using the NanoDrop 2000c spectrophotometer (Thermo Scientific GmbH, Dreieich, Germany). Templates were set to a DNA concentration of 0.1 ng/μl. DNA was stored at 6°C until use.

Later, a simple thermic cell lysis protocol (Ngamwongsatit et al., 2008) was used to enable a simplified DNA extraction method. The standardized DNA isolation with the DNEasy Blood and Tissue Kit was compared to this method based on bacterial cell dilution series of *B. cereus* DSM 31 as described in section “Bacterial Cell-Based Limit of Detection.” For this purpose, 1 ml of the cell suspension was centrifuged at 5,000 × *g* for 2 min, the supernatant was carefully discarded and the remaining cell pellet was washed in 500 μl TE-buffer (10 mM Tris-HCL, 1 mM EDTA, pH 8) (Sigma-Aldrich Chemie GmbH, Taufkirchen, Germany). After this, the centrifugation step was repeated and the supernatant discarded again. The washed cell-pellet was then homogenized with 100 μl TE-buffer and the suspension was boiled for 10 min. The boiled samples were centrifuged at 10,000 × *g* for 5 min to sediment the cell debris, and the DNA containing supernatants were collected, each in a new sterile tube, and stored at –20°C until use.

In a preliminary test, it was evaluated whether the DNA isolation method by boiling was also appropriate with matrix components in the template.

This method was finally used for all cell suspensions and enriched food matrices.

### Optimization of Loop-Mediated Isothermal Amplification Method-Reaction Temperature

For the selected primer sets, the reaction conditions were optimized regarding the reaction temperature. In accordance with the manufacturer’s recommendations, primer mixes were combined in a standard concentration as described in section “Reaction Approach and Preliminary Test,” and a concentrated version (F3/B3 0.2 μM, FIP/BIP 2.0 μM, LoopF/LoopB 1.0 μM). These were tested using a temperature gradient of 62–69°C and the temperature differed by 1°C from well to well. The reaction mixtures were pipetted as described in section “Reaction Approach and Preliminary Test.” All measurements were performed in triplicate. Detection time and amplification rate of the individual primer-temperature combinations were assessed.

### DNA Sensitivity

The DNA-based sensitivity was determined using the DNA decimal dilution series of *B. cereus* DSM 31. Using AE buffer (Qiagen GmbH), DNA concentrations of 10 ng/μl, 1 ng/μl, 0.1 ng/μl, 10 pg/μl, 1 pg/μl, 0.1 pg/μl, and 10 fg/l were used as template and investigated using both primer sets (*groEL, nheB*) and both primer concentrations. The reactions were carried out as described in section “Reaction Approach and Preliminary Test” using the evaluated reaction temperature as described in section “Optimization of Loop-Mediated Isothermal Amplification Method-Reaction Temperature.” Each dilution step was tested in triplicate. Detection times and the safe detection limit (100%) were used to evaluate the appropriate primer concentrations.

### Analytical Specificity

To determine the specificity of the reaction, a total of 40 *B. cereus* isolates, 57 isolates of the *B. cereus* group (excluding *B. cereus*) and 44 non-*B. cereus* group isolates were tested as shown in Table 2 and as described in section “Bacterial Strains and Culture Conditions.” The isolates originated from the institute’s own strain collection and were supplemented by strains from the German Collection of Microorganisms and Cell Cultures (DSMZ) and the Culture Collection of the University of Gothenburg (CCUG), Sweden. In addition, isolates were kindly provided by the co-authors. The species identity of these strains was confirmed by MALDI-TOF analysis.

DNA was extracted as described in section “DNA Extraction Method From Strains, Cell Suspensions and Enriched Food Matrices” and the concentration in the eluate was measured. All eluates were adjusted to a concentration of 0.1 ng DNA/μl.

To test all *Bacillus* spp. isolates for the presence of the *nheB* gene, the isolates were screened using a real-time PCR protocol as described in section “Real-Time PCR assays.”

### Bacterial Cell-Based Limit of Detection

One colony each of *B. cereus* DSM 31, DSM 4384 and field isolate MHI 3099 was inoculated into 10 ml buffered peptone water (Oxoid Deutschland GmbH) and enriched for 24 h at 37°C under aerobic conditions. After enrichment, a decimal dilution series was set up from 10^–1^ to 10^–9^ in accordance with DIN EN ISO 6887-1 (Anonymous, 2017) in 4°C cold buffered peptone water, and the dilution series was cooled at 4°C to prevent the pathogens from reproducing. To determine the bacterial count, 100 μl of dilution levels 10^–4^–10^–8^ were streaked out in duplicate on Mannitol-Egg Yolk-Polymyxin (MYP) agar (Oxoid Deutschland GmbH) in accordance with DIN EN ISO 7932 (Anonymous, 2004). The plates were incubated aerobically at 30°C for 24 h. After counting the MYP plates with 10–150 colonies, the number of colony-forming units was determined by the weighted arithmetic mean.

DNA of every dilution step was isolated using the simplified boiling method and in a short preliminary test compared to the Qiagen DNeasy Blood and Tissue Kit as described in section “DNA Extraction Method From Strains, Cell Suspensions and Enriched Food Matrices.” This experiment was performed in triplicate for each strain used.

### Real-Time Polymerase Chain Reaction Assays

A real-time PCR assay, also based on the *groEL* gene, was used as a comparative method to LAMP during the investigations of artificially contaminated food and the survey of natively contaminated food samples. RabalF-5′-GCAACTGTATTAGCACAAGCT-3′ and RabalR-5′-TTACC AACGCGCTCCATTGCTT-3′ were used as primers and the probe was 1-FAM-5′-GCTGCTATTTCTGCTGC TGACGAAGA-3′-BHQ1 (Lim et al., 2011). This real-time PCR assay was also performed using the LightCycler^®^ 96. Initial incubation took place at 95°C for 10 min, followed by 45 cycles of denaturation at 95°C for 15 s and annealing and synthesis at 60°C for 60 s. Each reaction batch contained 25 μl. These included 5.25 μl PCR grade water, 12.5 μl FastStart Essential DNA Probe Master Mastermix, 1 μl of each primer RabalF and RabalR (10 pmol/μl), 0.25 μl of the probe (25 pmol/μl) and 5 μl DNA template.

To test the isolates used and those found for the presence of the gene *nheB* and also to compare the samples, the following real-time PCR was used: Primers were *nheB*-3D-F 5′-GCA GCA GGR AAT ATT ATG-3′, *nheB*-3D-R 5′-GCT TTT GCT ACM GCA TGA AC-3′ and the probe was *nheB*-3D-Probe FAM-5′-AGC TGA AAG TAC AGT GAA ACA AGC TCC A-3′-BHQ1 (Abdulmawjood et al., 2019). Each reaction batch with a total volume of 25 μl contained 8.25 μl PCR gradient water, 12.5 μl FastStart Essential DNA Probes Master Mastermix (Roche Diagnostics GmbH, Mannheim, Germany), 0.75 μl *nheB*-3D-F and 0.75 μl *nheB*-3D-R primer (10 pmol/μl), 0.25 μl *nheB-*3D-Probe (25 pmol/μl) and 2.5 μl DNA-template. The amplification parameters consisted of initial denaturation at 95°C for 600 s, followed by 40 cycles including denaturation at 95°C for 10 s and annealing/synthesis at 60°C for 30 s (Abdulmawjood et al., 2019). This reaction was performed using the real-time PCR device LightCycler^®^ 96 (Roche Diagnostics GmbH). This real-time PCR was also used as comparison to the toxin gene targeting LAMP assay.

### Artificial Contamination of Food Matrices

The suitability of the LAMP assays for detecting the *B. cereus* group and the toxin gene *nheB* from the food matrix was tested by means of an inoculation experiment.

The cultural method, in accordance with DIN EN ISO 21871 (Anonymous, 2006), was used as a reference to LAMP. For cell counting, reference was made to DIN EN ISO Standard 7932 (Anonymous, 2004). Also, the previously described two real-time PCR assays were performed for comparison (see section “Real-Time PCR Assays”).

For this experiment, minced beef was first tested for the absence of presumptive *B. cereus* in accordance with the DIN standard. For this purpose, 10 g of the sample was weighed with 90 g buffered peptone water and homogenized for 2 min at 230 rpm using the Stomacher^®^ 400 Circulator (Seward Ltd., Worthing, United Kingdom). The remaining minced meat was stored at –20°C until use. A total of 1 ml of the initial dilution was transferred to 9 ml of single concentrated tryptone-soya-polymyxin B (TSP) broth (Oxoid GmbH) and homogenized. The tube was subsequently incubated aerobically at 30°C for 48 h. After the incubation period, 10 μl of the enriched broth was spread on MYP agar and on polymyxin pyruvate-yolk mannitol bromothymol blue (PEMBA) agar (Oxoid Deutschland GmbH). The agar plates were incubated aerobically at 30 and 37°C, respectively, for 24 h and then checked for suspicious colonies. On MYP agar, these formed 2–5 mm large, notched, pink colonies with a precipitation halo up to 5 mm in size on a crimson background. On PEMBA agar, they appeared similar, but in this case, the colonies and halo were bright blue with a green background. This coloration was enhanced when the plates were stored at room temperature for 24 h. Presumptive *B. cereus* were confirmed by detecting ß-haemolysis on COLS agar. For this, three suspect colonies were streaked out and the agar plate was incubated aerobically at 37°C for 24 h. The colonies were additionally confirmed by microscopic examination.

For a preliminary test, quantities of 10 g of minced beef were weighed and distributed to nine stomacher bags. Of these, one sample was retained as a negative control and not artificially contaminated. A decimal cell dilution series was prepared before using the strain *B. cereu*s DSM 31, as described in section “Bacterial Cell-Based Limit of Detection” and the cell content was determined. From this cell suspension, 1 ml was taken and used to contaminate one minced meat portion each time. The prepared samples were then made up to 100 g with buffered peptone water. The cell contents used are shown in Table 3. The samples and the buffered peptone water were homogenized for 2 min at 230 rpm and 1 ml each was taken for direct DNA isolation using the boiling method and 1 ml for further cultural examination. The bags were then incubated aerobically at 37°C for 3, 6, and 24 h and aliquots were taken again for DNA isolation and cultural examination at the respective time. The DNA was tested with both LAMP assays and real-time PCR assays. In parallel, cultural testing was performed as previously described to show whether the bacteria added to the sample could actually be grown again from the matrix and detected.

**TABLE 3:** Used cell contents for the preliminary test of DSM 31.

	Contamination level (CFU/10 g matrix)	CFU/g Matrix (Absolute)	CFU/ml weighing (Absolute)	CFU/reaction batch
NTC	0	0	0	0
1	0–1	0.00416	0.000416	0.000208
2	1–10	0.416	0.00416	0.00208
3	10–100	4.16	0.416	0.0208
4	100–1,000	41.6	4.16	0.208
5	1,000–10,000	416	41.6	2.08
6	10,000–100,000	4,160	416	20.8
**7**	**100,000–1,000,000**	**41,600**	**4,160**	**208**
**8**	**1,000,000–10,000,000**	**416,000**	**41,600**	**2,080**

*Bold, potentially critical cell counts; 8 $\hat{=}$ 4.16×10^6^ CFU/ml.*

A total of four *B. cereus* strains were selected for artificial inoculation in the main experiment: DSM 31, DSM 4384, MHI 3099, and additionally MHI 252 which was selected as another field strain to achieve a balance between reference and field strains. A total of three replicates with eight inoculation levels and one negative control were carried out per strain.

### Native Sample Analysis

A total of 100 samples were included in the investigations. These consisted of 80 raw meat products such as minced meat and also cooked samples such as meatballs and 20 different vegetarian substitute products (vegetarian sausage, burger patties, and similar products). In parallel, the qualitative detection as well as additional quantitative, cultural detection were carried out to assess whether the results from the artificial contamination tests correlated with those from the native sample tests.

All products were culturally tested for the presence of presumptive *B. cereus* in accordance with DIN EN ISO standard 21871. DNA isolation was performed by the boiling method after zero and 6 h, as already done for the artificially contaminated samples. The obtained DNA templates were checked using both LAMP assays and both real-time PCR assays. Presumptive *B. cereus* isolates obtained during the cultural investigation were also archived and confirmed by MALDI-TOF analysis after formic acid extraction.

### Simplified Colony-Confirmation Method

All isolates found were additionally subcultured on COLS agar (Oxoid GmbH) and colony material was collected using a 10 μl inoculation loop. DNA was isolated using a simplified boiling method. The colony material was stirred into 200 μl TE buffer (10 mM Tris-HCl, 1 mM EDTA, pH 8), homogenized and boiled for 10 min. A centrifugation step at 12,000 × *g* for 5 min followed to sediment the cell debris. The supernatant was used as a template for LAMP.

### Data Processing

Raw data analysis of LAMP was performed using the software GenieExplorer (OptiGene Ltd.) offered by the manufacturer of the used device Genie^®^ II. Calculations and graphics were generated using Microsoft^®^ Excel for Microsoft 365 MSO (Microsoft Corporation, Redmond, WA, United States).

## Results

### Preliminary Tests for the Selection of Suitable Primer Sets

The rejected primer set, *gyrB* (degenerate), showed cross-reactions with non-*B. cereus* group strains. In the second preliminary experiment, the remaining primer sets, *groEL* (non-degenerate and degenerate) and *gyrB* (non-degenerate), were tested on five further *B. cereus* isolates and three further *B. cereus* group isolates. All degenerate primers proved to be unsuitable, as the detection times were up to 10 min longer than those of primers without consideration of the mutants. In addition, when degenerate primers were used, some cross-reactions or no reactions took place within the run-time. Only the primer set for *groEL* (non-degenerate) was able to identify all tested isolates in the *B. cereus* group. Likewise, all strains tested in the preliminary test carrying the *nheB* gene were detected by *nheB*-LAMP. Based on these results, *groEL* and *nheB* primers were included in further assay optimization studies irrespective of the mutants.

### Simplified DNA Isolation Method

**FIGURE 1:**
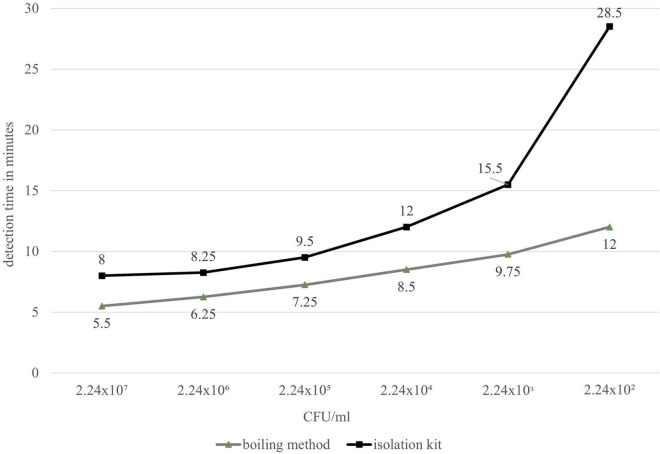
Comparative detection times of real-time fluorometer Genie^®^ II (LAMP) between DNeasy Blood and Tissue Kit (black) and the simplified boiling method by Ngamwongsatit et al. (2008) (gray).

Using a cell dilution series, the DNA obtained with the Qiagen DNeasy Blood and Tissue Isolation Kit and a simplified boiling method (Ngamwongsatit et al., 2008) was analyzed with the group-specific LAMP assay (*groEL*). With both methods, the last reliably detectable dilution level was 10^–5^, which had a cell content of 224 CFU/ml. Thus, the absolute detection limit of this assay in the preliminary test was 11.2 CFU/reaction approach regardless of the DNA isolation method. The decisive factor, however, was that the detection times using the boiling method were shorter than when using the kit (Figure 1). The boiling method was therefore used in the main experiment to determine the cell-based sensitivity and for all further experiments.

### Optimization of Loop-Mediated Isothermal Amplification Method-Reaction Conditions

**FIGURE 2:**
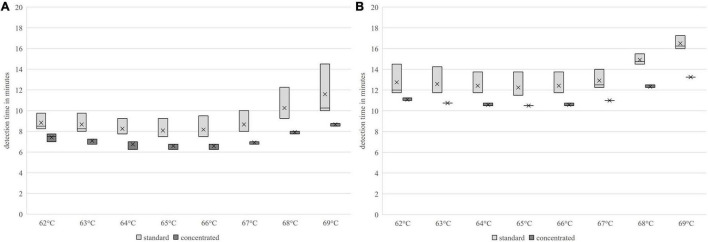
Comparison of the detection times using the LAMP method of different primer-temperature combinations; **(A)** *groEL*, **(B)** *nheB.*

The shortest detection times were achieved for both primer sets and concentrations in a temperature range of 65–66°C. The *groEL* primer set produced the earliest amplification signal in the concentrated version with $\bar{x}$ = 6.55 (*s* = 0.23) min at 66°C isothermal reaction temperature. The *nheB* primers produced an amplification signal at 65°C isothermal reaction temperature 1 min and 46 s earlier than the standard primer in the concentrated version with $\bar{x}$ = 10.47 min (*s* = 0). At 66°C, the detection time from the *nheB* standard primer was 7.8 s longer and that of the concentrated primer was 6 s longer. The results are presented in Figure 2. The amplification rate was higher for both primer sets with 66°C compared to 65°C, regardless of the concentration of the primers. The primer sets and their respective differently concentrated primer mix variants provided optimal reaction kinetics when considering the detection times and amplification rates at 66°C.

### Analytical Sensitivity and Primer Concentration Selection

The concentrated *groEL* primer was able to consistently detect 0.1 pg DNA/μl in all replicates. For the primer set in the standard concentration, the reliable detection limit was 1 pg DNA/μl. Therefore, the concentrated primer set for *groEL* was used for all subsequent experiments. The safe detection limit of the *groEL*-LAMP assay was 0.5 pg DNA.

For enterotoxin gene detection using *nheB*, a safe detection limit of 0.5 pg DNA per reaction batch was also determined; this, however, being for the standard concentrated primer set. A constant 0.1 pg DNA/μl could be detected. With the concentrated primer set, 1 pg DNA/μl was reliably detectable. The following investigations were therefore carried out using the *nheB* standard primer mix.

**FIGURE 3:**
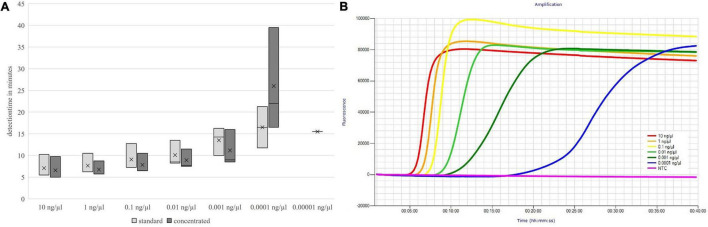
Detection times of both primer mix concentrations **(A)** and fluorescence signals of the concentrated primer mix **(B)** depending on the DNA concentration; *groEL.*

**FIGURE 4:**
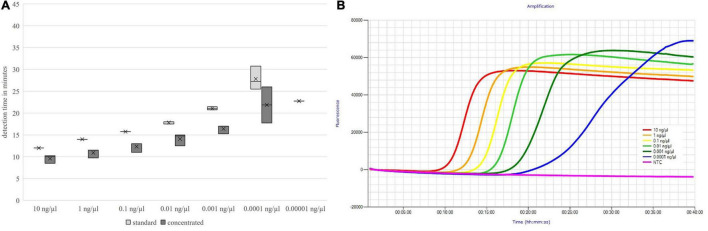
Detection times of both primer mix concentrations **(A)** and fluorescence signals of the standard concentrated primer mix **(B)** depending on the DNA concentration; *nheB.*

The detection times increased with decreasing DNA concentration, but up to the absolute detection limit, only a slight increase in the mean detection time could be seen. These results are shown in Figures 3, 4.

### Analytical Specificity

**FIGURE 5:**
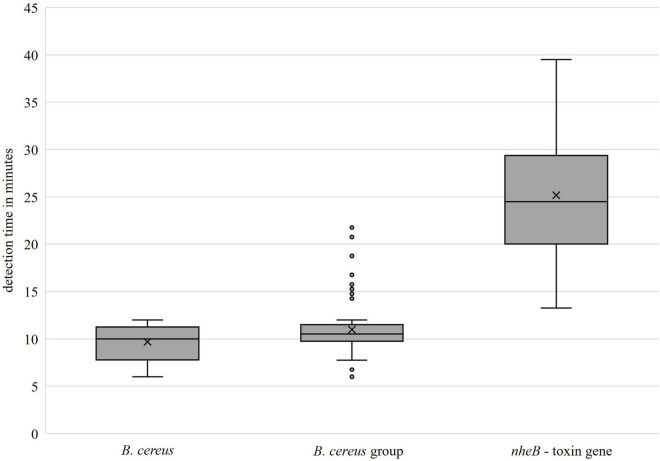
Detection times of the developed primer sets (*groEL*: left, center; *nheB:* right).

The primer set *groEL* for detecting the *B. cereus* group showed a specificity of 100% with respect to all tested *B. cereus* group isolates (including *B. cereus*) and non-*B. cereus* group strains. From a total of 96 isolates in the *B. cereus* group, 83 were endowed with the toxin gene *nheB*. Six isolates with *nheB* were not identified by the developed LAMP-*nheB*-assay, including all representatives of the *B. toyonensis* (five out of six) species. Two isolates were identified as false positives when the results of the real-time PCR used were taken as a reference. The primer set *nheB* was thus 93.75% specific. The positive and negative predictive values were calculated, the results of which are presented in Table 4. Figure 5 shows the detection times independent of the individual isolates. For the concentrated primer set *groEL*, the median value of the detection time was $\tilde{x}$ = 10 min (Q1 = 7.77 min, Q3 = 11.25 min) for *B. cereus* and $\tilde{x}$ = 10.5 min (Q1 = 9.75 min, Q3 = 11.5 min) for the *B. cereus* group, respectively. The toxin gene *nheB* was detected in the median after $\tilde{x}$ = 24.5 min (Q1 = 20.0 min, Q3 = 29.38 min), independent of the tested strain.

**TABLE 4:** Positive and negative predictive values.

	*PPV*	*NPV*
*groEL*	1	1
*nheB*	0.975	0.903

### Bacterial Cell-Based Limit of Detection

More than one *B. cereus* strain was selected in view of diversity of the isolates and the planned experiments on artificial contamination.

The last reliably detectable cell contents of the *groEL* assay were 13.6 CFU/reaction approach for DSM 31, 11.35 CFU/reaction for DSM 4384 and 27.05 CFU/reaction preparation for MHI 3099, respectively. Reliable detection was thus possible with initial contamination of 100–1,000 CFU/ml in the cell suspension. Direct detection in the homogenate of 10 g food could thus be expected from initial contaminations of 1,000–10,000 CFU/g food. The median detection time, independent of strain and cell content, was $\tilde{x}$ = 9.75 min (Q1 = 7.0 min, Q3 = 15.25 min).

For the assay for the detecting of the toxin gene *nheB*, the reliable detection limit for DSM 31 was 13.6 CFU/reaction, for DSM 4384 11.35 CFU/reaction and for MHI 3099 270.5 CFU/reaction preparation, respectively. These values show that the tested field isolate had a detection limit one log level higher than that of the reference strains. In the cell suspension, a reliable detection could be expected for initial contaminations of 1,000–10,000 CFU/ml, regardless of the strain. Direct detection in the homogenate of 10 g of any food sample could thus be expected from initial contamination levels of 10,000–100,000 CFU/g. The median detection time, independent of strain and cell content, was $\tilde{x}$ = 17.5 min (Q1 = 12.13 min, Q3 = 25.36 min).

Furthermore, the detection times of both assays were significantly longer for the field isolate MHI 3099.

### Analysis of Artificially Contaminated Food Samples

The preliminary test showed that a direct detection of the critical value (cell contents from 10^5^ CFU/g food) was possible using both LAMP assays and *nheB*-real-time PCR. After a 3-h incubation period at 37°C, there was no improvement in the detection limit. After a 6-h incubation period, up to 41.6 CFU/g food could be detected as initial contamination by LAMP. After 24 h, all levels were detectable. Culturally, however, a clear identification of the presumptive *B. cereus* was not always possible after 24 h. Various pathogens of the background microbiota, e.g., Enterococci or *Proteus* sp., had grown on the selective plates and displaced *B. cereus*. For this reason, the sample was examined in the following experiments both without enrichment and with a short enrichment period of 6 h. Hereby, the assays were adapted as rapid test procedures in the direct procedure or with results on the same day.

An extra preliminary test was performed to evaluate whether the boiling method for DNA isolation was also possible with matrix components in the template, as described in section “DNA Extraction Method From Strains, Cell Suspensions and Enriched Food Matrices.” Both assays were able to detect an initial contamination of 100–10,000 CFU/ml. Thus, incubation times of zero and 6 h were selected and the simplified DNA isolation method using thermal cell disruption was chosen.

The main experiment showed that direct detection of the critical value was possible in 10 out of 12 artificially contaminated samples using *groEL*-LAMP. The two samples that tested negative were contaminated with the field isolate MHI 3099. Reliable detection of the critical value (from 10^5^ CFU/g food) of *B. cereus* group representatives was possible in all 12 samples after 6 h of enrichment. The initial contamination level was 3.27 × 10^5^–8.5 × 10^5^ CFU/g food. Initial contaminations which were one log level lower were also directly detectable in 10 out of 12 samples. The two samples which tested negative were again contaminated with the field strain MHI 3099. With the comparative *groEL*-real-time PCR, an initial contamination of 3.27 × 10^5^–8.5 × 10^5^ CFU/g was only detectable in nine out of 12 sample after 6 h of enrichment. Direct detection was not clearly possible.

Using *nheB*-LAMP, direct detection of the critical value was only possible in 50% of the cases. After 6 h of enrichment, 11 out of 12 samples were positive. The one sample that tested negative was also from the field isolate MHI 3099. The *nheB*-real-time PCR was able to reliably detect an initial contamination of 3.27 × 10^5^–8.5 × 10^5^ CFU/g already in direct detection. After 6 h of enrichment, initial contamination of 3.27 × 10^3^–8.5 × 10^3^ CFU/g could be reliably detected.

By means of cultural, qualitative detection, suspect colonies could be reliably confirmed in direct detection at initial contamination levels of 3.27 × 10^3^–8.5 × 10^3^ CFU/g. After 6 h of enrichment, this was the case for initial contaminations of 3.27 × 10^1^–8.5 × 10^1^ CFU/g.

Those samples not recognized as positive by the field strain MHI 3099 reduced the quality of the detection rate. This strain had already been characterized by significantly longer detection times in the investigations to determine sensitivity on a cell basis. In addition, this strain showed rather atypical colonies for *B. cereus* in the cultural examination and was more difficult to enrich. In the tests to determine specificity, eight of the 96 *B. cereus* group isolates tested showed longer detection times (see Figure 5).

The results are presented in Table 5.

**TABLE 5:** Results of the artificial contamination tests; bold: critical value and one log level below.

	0 H enrichment						

	Contamination level (CFU/10 g matrix)	CFU/g template (absolute)	LAMP *groEL*	LAMP *nheB*	qPCR *groEL*	qPCR *nheB*	Cultural
DSM31, DSM 4384,	0	0	0/12	0/12	0/12	0/12	0/12
MHI 3099, MHI 252	0–1	0.03 (alte) l	0/12	0/12	0/12	0/12	0/12
	1–10	0.33 (alte) l	0/12	0/12	0/12	0/12	1/12
	10–100	3.27 (alte)	0/12	0/12	0/12	0/12	1/12
	100–1,000	32.7 alte	0/12	0/12	0/12	0/12	5/12
	1,000–10,000	327 0 alte	1/12	0/12	0/12	2/12	10/12
	10,000–100,000	3.27 × 10^3^ 02005 × 10^3^	3/12	1/12	2/12	2/12	12/12
	**100,000–1,000,000**	**3.27**×**10^4^ 020,5 × 10^4^**	**7/12**	**2/12**	**1/12**	**8/12**	**12/12**
	**1,000,000–10,000,000**	**3.27**×**10^5^ 02005**×**10^5^**	**10/12**	**6/12**	**4/12**	**12/12**	**12/12**

	**6 H enrichment**						

	**Contamination level (CFU/10 g matrix)**	**CFU/g template (absolute)**	**LAMP *groEL***	**LAMP *nheB***	**qPCR *groEL***	**qPCR *nheB***	**Cultural**

DSM31, DSM 4384,	0	0	0/12	0/12	0/12	0/12	0/12
MHI 3099, MHI 252	0–1	0.03 (alte) l	0/12	0/12	0/12	0/12	0/12
	1–10	0.33 (alte) l	2/12	1/12	0/12	1/12	4/12
	10–100	3.27 (alte)	6/12	3/12	2/12	4/12	7/12
	100–1,000	32.7 alte	9/12	3/12	3/12	8/12	12/12
	1,000–10,000	327 0 alte	9/12	6/12	4/12	10/12	11/12
	10,000–100,000	3.27 × 10^3^ 02005 × 10^3^	9/12	8/12	7/12	12/12	12/12
	**100,000–1,000,000**	**3.27**×**10^4^ 020,5**×**10^4^**	**10/12**	**9/12**	**8/12**	**12/12**	**12/12**
	**1,000,000–10,000,000**	**3.27**×**10^5^ 02005**×**10^5^**	**12/12**	**11/12**	**9/12**	**12/12**	**12/12**

*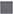 100–90% 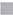 80–50%*

*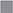 90–80% 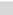 <50% Negative*

### Native Sample Analysis

A total of 100 food samples were analyzed. The results of the investigation are shown in Table 6. A total of 63 food isolates of *B. cereus* group isolates were collected. Of these, 59 were toxin gene carriers, these being determined by using *nheB* real-time PCR.

**TABLE 6:** Results of native sample analysis (*n* = 100).

Contamination level (CFU/g), 0 h	Cultural positive	*groEL* LAMP	*groEL* qPCR	*nheB* LAMP	*nheB* qPCR	MALDI +
>10^0^–10^1^	12	1	1	0	2	12
10^1^–10^2^	1	0	0	0	0	1
10^2^–10^3^	1	1	1	0	0	1
10^3^–10^4^	2	2	2	0	1	2
10^4^–10^5^	0	0	0	0	0	0

10^5^–10^6^	1	1	0	0	0	1
Total positive:	17	5	4	0	3	17*
Add. false positive:	–	0	0	0	2	

**Contamination level (CFU/g), 6 h**	**Cultural positive**	***groEL*** ** LAMP**	***groEL*** ** qPCR**	***nheB*** ** LAMP**	***nheB*** ** qPCR**	**MALDI +**

>10^0^–10^1^	6	0	2	0	1	6
10^1^–10^2^	0	0	0	0	0	0
10^2^–10^3^	8	2	2	0	1	8
10^3^–10^4^	20	5	6	2	6	20
10^4^–10^5^	10	7	5	1	5	10

10^5^–10^6^	2	2	1	2	1	2
Total positive:	46	16	16	5	14	46**
Add. false positive:	–	0	1	0	5	

*1 isolate of B. thuringiensis, **2 isolates of B. thuringiensis, gray, critical value (from 105 CFU/g food).

### Boiling-Method of Bacterial Colonies

The isolates found were analyzed using the established rapid method for detection of colony material. All 63 isolates in the *B. cereus* group were detected using the *groEL* LAMP. A total of 55 of the 59 isolates carrying the toxin gene *nheB* were detected using the developed toxin LAMP assay.

Accordingly, the specificity of the *groEL* LAMP assay was 100% and that of the toxin gene assay (*nheB*) 93.22%.

## Discussion

A LAMP method for specific and rapid detection of critical cell counts of potentially non-haemolytic enterotoxin-producing *B. cereus* is difficult to implement because of the high degree of relatedness of the members of the *B. cereus* group (Carroll et al., 2020). To achieve the best possible detection of relevant representatives of the *B. cereus* group, a two-step assay was developed detecting all representatives of the current *B. cereus* group and, in a second step, a toxin gene. As group-specific target gene, *groEL* (Park et al., 2007; Lim et al., 2011) was chosen after the described preliminary tests.

An assay for the emetic type, targeting *cesA* gene has already been established (Liu et al., 2010). Therefore, it was decided to focus on the enteropathogenic type. For the identification of potentially enteropathogenic isolates, the genes encoding the three main known enterotoxins were considered. Cytotoxin K, encoded by the *cytK* gene, proved unsuitable as representative target gene. First, the highly conserved variant *cytK-1* occurs only in a few highly toxic strains which were reclassified as *B. cytotoxicus* (Ehling-Schulz et al., 2006; Guinebretière et al., 2013). Second, the more common variant *cytK-2* occurs in approx. 40–70% of all tested isolates (Dietrich et al., 2021), but the role of its corresponding protein in the diarrhoeal disease seems to be rather negligible (Fagerlund et al., 2004; Ngamwongsatit et al., 2008). The *hbl* operon, which encodes the three-component enterotoxin complex haemolysin BL is also rather unsuitable, as only approx. 40–70% of all isolates bear those genes (Jeßberger et al., 2015; Dietrich et al., 2021). Thus, the *nhe* operon was chosen. The genes *nheA*, *nheB* and *nheC* encode the protein toxin components NheA, NheB, and NheC, respectively. NHE is recognized as relevant virulence factors causing diarrhoeal disease (Dietrich et al., 2021). As earlier studies showed that cytotoxic activity correlates with the amount of secreted NheB protein (Moravek et al., 2006; Jeßberger et al., 2015), the *nheB* gene was chosen as target.

All degenerate primers were found to be unsuitable. The use of degenerate primers sometimes led to cross-reactions in which the primers bound to several sequences or no reactions took place within the run-time. Only the primer set for *groEL* (non-degenerate) was able to identify all tested isolates of the *B. cereus* group. Likewise, all strains carrying the *nheB* gene were detected by *nheB*-LAMP in the preliminary test. Based on these results, *groEL* and *nheB* primers were included in further assay optimization studies without considering the mutants.

The specificity of the developed LAMP assay was 100% for *B. cereus*-group isolates and 93.7% for detecting *nheB.* Other studies tested their specificity, e.g., for *cesA*, only on 19 *B. cereus* strains (Liu et al., 2010) or even fewer representative selections of strains (Deng et al., 2019). The present study showed specificity on 97 *B. cereus* group isolates, which allows a more precise statement. Both primer sets showed an analytical sensitivity of 0.1 pg DNA/μl. Other studies determined sensitivity based only on cell counts per microlitre and not on pure DNA dilution series (Deng et al., 2019). Basically, almost no assays have been developed so far using the approach followed in this study. Using simplified DNA extraction by boiling allows a suitable detection of bacterial cells for both developed assays. The boiling method proved to be more suitable, as shown in Figure 1. This can be explained by the fact that more DNA could be obtained with the boiling method. Due to the extracellular matrix of the *Bacillus* spp. isolates, the membrane of the spin column used in the Qiagen DNeasy Blood and Tissue Kit could become clogged and not all DNA molecules could be washed out.

When optimizing the reaction temperature, a uniform temperature of 66°C was finally chosen, so that it was possible to test for the *B. cereus* group and *nheB* toxin carriers simultaneously in one run of the Genie^®^ II. Since the concentrated primer mixtures had only produced a fluorescence signal about 1–1.5 min earlier, both primer concentrations were included in the determination of sensitivity based on a DNA decimal dilution series, and only then were the final assay parameters determined taking into account the primer concentrations.

The last reliably detectable cell contents of the *groEL* assay were one log level lower for one field strain than for the other tested isolates. These results can be inferred from the fact that *B. cereus* has a high genetic diversity, and the target gene sequences in the field isolates may show deviations compared to the reference strains used for the primer design. The assay for detecting the *nheB* toxin gene showed similar results.

Usually, a cell content of 10^5^ CFU/g food is often given as a threshold value (Rajkovic et al., 2008; Stenfors Arnesen et al., 2008; Dietrich et al., 2021) even if lower cell levels could already trigger symptoms. This corresponds to a content of 10,000 CFU/ml homogenate or 500 CFU/reaction preparation. There can also be a risk of disease at levels of around 10^3^ CFU/g and below, but this appears less frequently (BfR, 2020a). Thus, the assessment of the assay was oriented toward the guideline value of 10^5^ CFU/g (critical value). From the results of the artificial contamination, it could be deduced that the large diversity of the *B. cereus* group did influence the quality of the results. Therefore, it can be assumed that a deterioration in the detection limit is to be expected in approx. 9% of the cases. This can be explained by the high heterogeneity of the *B. cereus* group.

In contrast to *groEL*-real-time PCR, *groEL*-LAMP can reliably detect cell contents in the range of the critical value. This is also possible with the cultural method, but the results are available on the same day using LAMP and only after 3 days using the cultural method. If more specific analyses are to follow, then results take even longer. It is a decisive advantage to receive the results within 1 day using the developed LAMP method.

The toxin gene LAMP for *nheB* is not as sensitive as the comparative real-time PCR but allows conclusions to be drawn about the content of toxin gene-bearing *B. cereus* even after 6 h of enrichment. Other assays developed were not able to provide information on the content of relevant *B. cereus*, or only to a limited extent or with a different approach (Liu et al., 2010; Deng et al., 2019).

With the developed two-step assay, an application in food is possible under simple conditions. Even without enrichment, a statement can be made as to whether a food contains potentially relevant amounts of representatives of the *B. cereus* group. So far, the assay has mainly been tested with cells. However, the results with the native samples suggest that the assay could also be suitable for spores, as it cannot be safely assumed that vegetative cells of *B. cereus* were present in all samples. After 6 h of enrichment or also by means of the robust method of isolating DNA from colony material, the second LAMP assay can be used to determine whether the detected members of the *B. cereus* group have the genetic prerequisites to produce the non-haemolytic enterotoxin and thus possibly also cause diarrhoeal diseases.

## Conclusion

After successful establishment of the LAMP assay for the detection of isolates of the *B. cereus* group and *nheB* gene, its applicability in food was demonstrated. The extensive tests on a large number of different isolates, artificially contaminated and native samples, were able to show that detection was possible with the help of the two-step LAMP assay. It is particularly noteworthy that among 100 natively contaminated food samples, the relevant contamination levels could be reliably detected with the help of *groEL*-LAMP and these within 24 h. Reference methods such as cultural detection require significantly more work and time. The real-time PCR used for comparison did not show such reliable detection rates. The detection of the nheB gene was also successful. Thus, it can be concluded that with the *groEL/nheB*-LAMP assay, a possibility of detecting critical amounts of presumed toxin-producing cells of the *B. cereus* group was developed within 1 day. Both assays are also ideally suited for rapid screening of colony material on selective media or blood agar plates. In future studies, further investigations can be carried out based on the developed assays. For example, the ability to detect spores would be another approach that could be pursued, as the uptake of spores from contaminated food also plays an important role.

By means of the developed assay, a suitable and reliable basis was created for this.

## Data Availability Statement

The original contributions presented in the study are included in the article/supplementary material, further inquiries can be directed to the corresponding author/s.

## Author Contributions

AB performed the experiments and wrote the manuscript draft. AB, US, and AA designed the experiments NJ and HF provided resources for the study. MP coordinated the research. AA was responsible for project administration and funding acquisition. All authors proofread and edited the manuscript.

## Conflict of Interest

The authors declare that the research was conducted in the absence of any commercial or financial relationships that could be construed as a potential conflict of interest.

## Publisher’s Note

All claims expressed in this article are solely those of the authors and do not necessarily represent those of their affiliated organizations, or those of the publisher, the editors and the reviewers. Any product that may be evaluated in this article, or claim that may be made by its manufacturer, is not guaranteed or endorsed by the publisher.

## Footnotes

1
https://blast.ncbi.nlm.nih.gov/Blast.cgi

2
http://primerexplorer.jp/lampv5e/index.html
